# Calmodulinopathy: Functional Effects of CALM Mutations and Their Relationship With Clinical Phenotypes

**DOI:** 10.3389/fcvm.2018.00176

**Published:** 2018-12-11

**Authors:** Beatrice Badone, Carlotta Ronchi, Maria-Christina Kotta, Luca Sala, Alice Ghidoni, Lia Crotti, Antonio Zaza

**Affiliations:** ^1^Department of Biotechnology and Bioscience, University of Milano-Bicocca, Milan, Italy; ^2^Center for Cardiac Arrhythmias of Genetic Origin and Laboratory of Cardiovascular Genetics, Istituto Auxologico Italiano, IRCCS, Milan, Italy; ^3^Department of Medicine and Surgery, University of Milano-Bicocca, Milan, Italy; ^4^Department of Cardiovascular, Neural and Metabolic Sciences, San Luca Hospital, Istituto Auxologico Italiano, IRCCS, Milan, Italy

**Keywords:** calmodulin mutations, ion channels, repolarization, Ca^2+^ handling, arrhythmia mechanisms

## Abstract

In spite of the widespread role of calmodulin (CaM) in cellular signaling, CaM mutations lead specifically to cardiac manifestations, characterized by remarkable electrical instability and a high incidence of sudden death at young age. Penetrance of the mutations is surprisingly high, thus postulating a high degree of functional dominance. According to the clinical patterns, arrhythmogenesis in CaM mutations can be attributed, in the majority of cases, to either prolonged repolarization (as in long-QT syndrome, LQTS phenotype), or to instability of the intracellular Ca^2+^ store (as in catecholamine-induced tachycardias, CPVT phenotype). This review discusses how mutations affect CaM signaling function and how this may relate to the distinct arrhythmia phenotypes/mechanisms observed in patients; this involves mechanistic interpretation of negative dominance and mutation-specific CaM-target interactions. Knowledge of the mechanisms involved may allow critical approach to clinical manifestations and aid in the development of therapeutic strategies for “calmodulinopathies,” a recently identified nosological entity.

## Introduction

As other ions, Ca^2+^ is used as a charge carrier to modulate membrane potential; however, Ca^2+^ also has a central role as a diffusible signaling molecule and as a trigger of diverse cellular functions. While some of these are clearly complementary in achieving a functional goal (e.g., cAMP signaling in functional upregulation) and can coexist, others are devoted to apparently competing aims (e.g., apoptosis pathway) and need to be separated. This requires mechanisms allowing intracellular Ca^2+^ to act on its targets with high specificity. Several strategies are employed by the cell to achieve this goal. Ca^2+^ buffering by intracellular proteins and small molecules, leads to a strictly controlled mobility of the ion. Active Ca^2+^ compartmentalization within organelles allows to keep “resting” Ca^2+^ concentration in the general (or “bulk”) cytosolic compartment at very low levels (around 10^−7^ M), i.e., below the threshold required to activate downstream effectors; at the same time, structural organization (e.g., T-tubules) allows very small Ca^2+^ fluxes to achieve high Ca^2+^ concentration in the specific subcellular compartment hosting the target effector (1).

The presence of molecules devoted to detect Ca^2+^ and transduce its concentration changes into specific actions is a further strategy, pivotal to the integrated operation of Ca^2+^-dependent processes. Perhaps the most diffuse of such “Ca^2+^ sensor” molecules is calmodulin (CaM), a protein present in all cell types and highly conserved throughout evolution (1). Most Ca^2+^-binding proteins are characterized by “EF hand” domains, which constitute the ion binding site. Whereas, in proteins involved in Ca^2+^ buffering and controlled mobility the EF hand is simply a binding site, in Ca^2+^ sensors the EF domain changes protein conformation in response to Ca^2+^ binding, thus triggering a variety of downstream events (2).

Essential to CaM's targeting role, is its property to stably bind to many of its downstream effectors. This corresponds to the presence on the latter of specific CaM-binding sequences, which make CaM an integral component of the target protein. Thus, CaM can exist as a freely diffusible signal (cytosolic pool), or as a sensor intrinsic to a given effector (bound pool), thus affording either diffuse or highly confined signaling. Furthermore, in various cells types, the cytosolic CaM pool can be redistributed to the nuclear compartment upon a rise in Ca^2+^ levels, thus broadening the targets range (3, 4). Also, except in selected cell types (e.g., mitotic cells), local CaM concentrations may follow Ca^2+^ oscillations, thus generating spatial and temporal patterns which may play a crucial role in biological processes (5).

In keeping with its central and evolutionarily conserved function, CaM is generated in a highly redundant mode. Indeed, an identical amino acid sequence is encoded by 3 CaM genes (CALM 1, 2, and 3) (5). Such redundancy is in apparent contrast with the high penetrance of heterozygous CaM mutations. While the possible role of transcriptional regulation of these genes is discussed in the accompanying article (6), specific molecular mechanisms may contribute to negative dominance in a mutation's effect.

In cardiac muscle cells, Ca^2+^ at the same time contributes as a charge carrier to electrical excitation (the “action potential,” AP) and triggers the development of mechanical force; therefore, Ca^2+^ is crucial to excitation-contraction coupling (ECC) (7). Several processes central to beat-to beat control of intracellular Ca^2+^ dynamics are CaM-mediated; furthermore, CaM acts a Ca^2+^ sensor in the control of gene expression, thus playing a role in long-term modulation of cell function and fate (8). This might lead to the expectation that a CaM loss of function should result in general myocyte dysfunction and death. However, this is contradicted by the observation that CaM mutations affect only the function of specific targets, leading to mutation-specific phenotypes with pronounced electrical instability as a common feature (9).

Our objective is to revise the information available on the various aspects of CaM structure/function that we see as potentially relevant to a mechanistic interpretation of CaM mutations phenotypes, with a focus on cardiac ones.

### CaM Structure, Ca^2+^-Sensing and Target Recognition

CaM is composed of 149 amino acid residues to form a 17 kDa protein. The protein is ubiquitous and expressed in all eukaryotic cells, with 100% identity in its amino acid sequence among vertebrates. Three genes (CALM1, CALM2, and CALM3) encode CaM with an identical amino acid sequence, thus resulting in potential redundancy (10).

**Figure 1:**
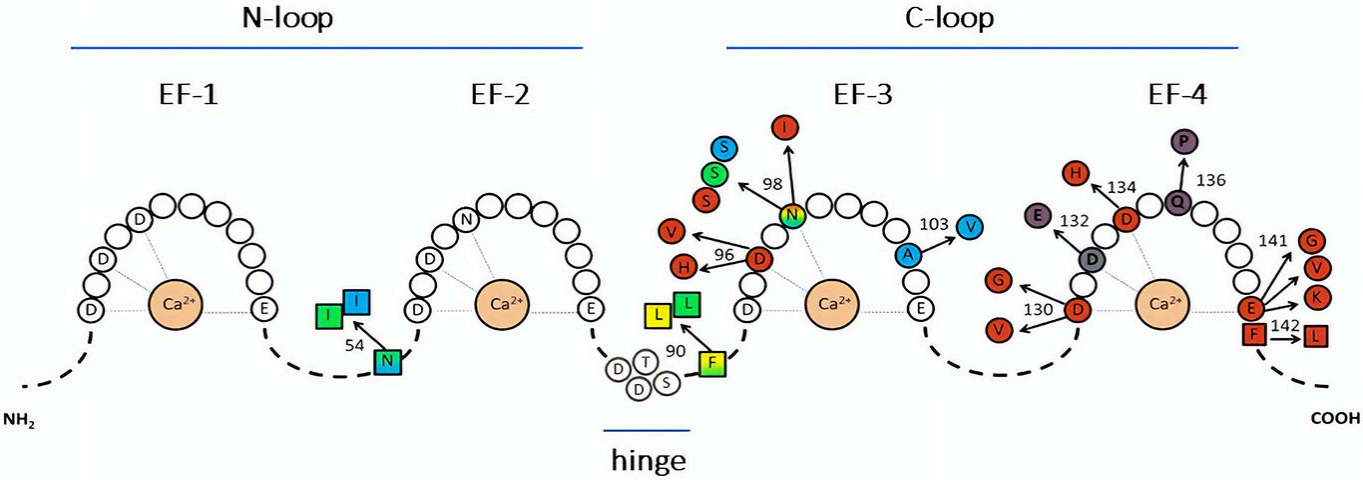
Representation of CaM sequence and relative disease-associated mutations. The letters identify amino acids directly involved in Ca^2+^ binding (within the EF-hands), or in the hinge region. Color-substituted amino acids represent mutations in the EF-hands (circles) or in the linkers (squares); colors correspond to the associated phenotype: catecholaminergic polymorphic ventricular tachycardia (CPVT, light blue), long QT syndrome (LQTS, red), idiopathic ventricular fibrillation (IVF, yellow), other unexplained sudden death (green). LQTS/CPVT overlap mutations are shown in shaded color. Modified from Crotti and Kotta, (11).

CaM is formed of two “lobes,” named N and C, respectively, according to their position with respect to protein ends, connected by an α-helix “linker” containing a flexible region (“hinge”) (Figure 1) (12); this allows each lobe to move relative to the other. Each lobe consists of two “EF hand” domains (EF) with one Ca^2+^ binding site each (2 Ca^2+^ binding sites per lobe). All EF hand domains can bind Ca^2+^; however, while the N-lobe (EF I and EF II) has higher affinity for Mg^2+^, the C-lobe (EF III and EF IV) binds Ca^2+^ with ten times higher affinity (13). Another functional distinction between the two CaM lobes is the rate of Ca^2+^ binding and unbinding, faster for the N-lobe than for the C-lobe (14).

Knowledge of CaM's 3D structure has evolved since its first description in 1985 (15), with a major contribution provided, 10 years later, by nuclear magnetic resonance (16). Whereas, this technology revealed detail of the linker helix accounting for its flexibility (17, 18), more recent studies added information about recognition of target proteins and how it can be affected by CaM complexing with Ca^2+^ (12, 19).

The interplay between structure and function is relevant to the three components of CaM signaling: Ca^2+^ binding, target binding, and target modulation.

The first aspect of interest is CaM's interaction with Ca^2+^. In apo-CaM (the Ca^2+^ unloaded CaM), EF I and EF II (N-lobe) are in a packed conformation, thus with low affinity for Ca^2+^; EF III and EF IV (C-lobe) are instead in a partially open conformation, more prone to Ca^2+^ binding. At resting Ca^2+^ levels (about 10^−7^ M), all CaM binding sites are typically unoccupied. When Ca^2+^ rises, its binding to the C-lobe sites triggers a conformational change leading the N-lobe to increase its affinity for Ca^2+^. In other words, Ca^2+^ binding to CaM is “cooperative,” i.e., the overall affinity increases when Ca^2+^ concentration exceeds the threshold for C-lobe occupation. CaM can potentially bind 1–4 Ca^2+^ ions; activation of downstream targets requires CaM being loaded with 3 Ca^2+^ ions at least (12), a configuration referred to as “holo-CaM.”

Also of interest are the structural aspects of CaM's interaction with target proteins, which are necessary for modulation of their activity. “Anchor” domains on target proteins are characterized by hydrophobic residue sequences flanked by negatively charged ones; the latter provide electrostatic interaction that may orient CaM binding (12). Many CaM targets (e.g., Ca_v_1.2) are characterized by the presence of a typical basic amino acid sequence called the “IQ motif.” The IQ motif is closely preceded by a “preIQ” region, which is a common site of permanent CaM binding (20). CaM interaction with the IQ motif itself is instead more likely responsible for downstream signaling (8, 14); indeed, specificity of the IQ sequence may dictate whether Ca^2+^-CaM signaling leads to target activation or inhibition (20). A well-studied instance of preIQ-IQ binding is CaM interaction with the voltage-dependent L-type Ca^2+^-channel (Ca_v_1.2); the detailed mechanism and Ca^2+^-dependency of this specific interaction will be described in paragraph Voltage-gated Ca^2+^ channels (Cav1.2).

As for Ca^2+^ sensing, CaM domains mainly involved in target binding are the lobes. Both CaM lobes contain nine methionine residues, playing a key role in target recognition, whose high flexibility provides plasticity crucial for this function (21).

The CaM binding interface is highly structured, but it may dynamically accommodate various binding modes in a metastable equilibrium. Ca^2+^ may stabilize a given interface conformation (12). Ca^2+^-dependency of CaM binding differs among target proteins. While holo-CaM is strictly required for strong target binding in some cases, in others target interaction occurs preferentially at lower levels of occupancy by Ca^2+^. This accounts for the finding that CaM may be bound to many targets at resting Ca^2+^ concentrations (8, 12, 22). At low Ca^2+^ the N-lobe has higher stability than the C-lobe (23), which favors its binding in the apo form (pre-association) (8). On the other hand, due to its higher Ca^2+^ affinity, the C-lobe is more often involved in Ca^2+^-dependent target binding than the N-lobe (12, 24) and both the N- and C-lobes may participate to stabilize the protein-target complex (25).

Ca^2+^ binding changes CaM conformation to expose hydrophobic (methionine-rich) sites, either at the N-lobe or at the C-lobe, suitable to interact with hydrophobic residues in the anchor (18). The large size and flexibility of CaM interaction landscape is essential to accommodate a variety of “anchor” side-chains, thus providing CaM with its extraordinary pleiotropicity. Nonetheless, the observation that single site mutations may selectively affect CaM binding to a specific target suggests high specificity of the binding interface. Less information exists about the target binding mode of apo-CaM. The main differences with holo-CaM binding may concern the N-lobe and a larger involvement of electrostatic interactions (12).

Consistent with the notion that CaM lobes change their conformation when interacting with the target, the relationship between target and Ca^2+^ binding by CaM is mutual: binding to the target may increase CaM affinity for Ca^2+^ (12); this generates cooperativity in Ca^2+^-dependency of CaM-target interactions. According to modeling data (26), differences among targets in the extent of such cooperativity contribute to specificity of target recognition.

The third aspect of CaM signaling is modulation of target function which, depending on the target, can be either stimulatory or inhibitory. Target modulation can be either enacted by apo-CaM, or require Ca^2+^ binding and its mechanism differs among targets (see below examples for RyR and Ca_v_1.2 channels). As a general interpretational scheme, CaM binding may stabilize an otherwise short-lived configuration, spontaneously assumed by the target protein and associated with a specific functional state (12).

### “Free” and “Pre-Bound” CaM Pools

Many CaM targets have been described so far. These include proteins involved in cell cycle, cell proliferation and autophagy (in healthy cells), tumor progression proteins (in cancerous cells) (5), proteins essential to cell communication and metabolism (27), and a wide number of ion channels (28–30). Most of these targets strictly require CaM pre-association to allow their regulation (31).

To better understand the role of CaM in cells, we need to consider its distribution between the “free” (cytosolic) and “pre-bound” pools. As mentioned previously, such a distribution may vary according to CaM's occupancy by Ca^2+^ (25).

Total CaM concentration is variable among tissues: it usually ranges between 5 and 40 μM (e.g. ,about 6 μM in intact myocytes) (32), but values up to 100 μM have been described in specific cell types (e.g., in the testis) (33). In cardiomyocytes, even at diastolic Ca^2+^ concentration, 99% of total intracellular CaM is bound to cellular proteins, leaving about 50-100 nM of free CaM (1%) in the cell (32). Nonetheless, the proportion of CaM in the pre-bound pool is variable among tissue types (e.g., 11% in testis and 63% in spleen), and it may differ between normal and pathological cells and depend on environmental factors, such as cell density in culture (5). The pre-bound CaM pool includes mainly apo-CaM or CaM with incomplete occupancy, depending on the target (32); its functional relevance may be to increase speed of target response to local Ca^2+^ elevation.

The pre-bound CaM pool localizes to structures in the plasma membrane as well as in intracellular organelles; under resting Ca^2+^ concentrations (e.g., 100 nM), pre-bound CaM may largely exist as apo-CaM. Pre-bound apo-CaM may even be released to the free CaM pool in response to Ca^2+^ elevation, thus representing a local diffusible CaM store (22); this is true particularly in growing cells, where CaM is highly expressed.

As Ca^2+^ occupancy increases, CaM becomes almost completely bound to targets, thus leaving a very small pool of freely diffusible holo-CaM. Competition among targets for this pool may be of significance for their reciprocal regulation (32). Even if the CaM-target interaction is generally facilitated by Ca^2+^, the pattern is quite complex. Chin and Means (22) identified at least six CaM target groups, according to their CaM recognition sequences and Ca^2+^-dependency of binding. Some of them are strongly pre-bound to apo-CaM, for others binding is stronger for holo-CaM, while, finally, apo-CaM binding to a class of targets can be released by Ca^2+^, thus providing a local reservoir for free CaM.

## CaM Modulation of Voltage-Gated Channels

### Voltage-Gated Ca^2+^ Channels (Ca_v_1.2)

Ca^2+^ current from Ca_v_1.2 channels (I_CaL_) is the most abundant type in cardiomyocytes (34). The symbol “L” recapitulates the main features of this channel (as compared to other Ca^2+^ ones): large conductance, activation at larger depolarizations and long lasting openings. The activation of this channel is driven by the action potential upstroke, with I_CaL_ reaching a peak in 2–7 ms. Thereafter, channels inactivate with time constants in the order of 50–100 ms at plateau potential, due to both Ca^2+^ and voltage-dependent gating (35). Ca^2+^ influx through I_CaL_ leads to a rapid increase of cytosolic Ca^2+^ concentration in the confined space between the sarcolemmal and sarcoplamic reticulum (SR) membranes (also called “dyadic cleft”). This is responsible for the opening of ryanodine receptors (RyRs), Ca^2+^-activated Ca^2+^channels clustered in the SR membrane facing Ca_v_1.2 channels.

I_CaL_ is modulated by two feedback signals that, albeit of opposite sign, are both dependent on the rise of Ca^2+^ concentration close to cytosolic mouth of the channel and involve CaM. Ca^2+^-dependent inactivation (CDI) is responsible for most of the rapid I_CaL_ decay occurring during sustained depolarization (35). Ca^2+^-dependent facilitation (CDF), a weaker phenomenon, reflects instead the increase in I_CaL_ peak conductance that may be observed during repetitive activation at high rates (36). Both CDF and CDI depend on Ca^2+^-CaM complexing (37). Earlier studies (37) reported that, whereas replacement of isoleucine or glutamine to alanine in the IQ motif of the C-terminal region of the Ca_v_1.2 channel α_1C_ subunit abolished CDI but enhanced CDF, replacement of isoleucine to glutamate in the same region abolished both forms of auto-regulation. This led to the conclusion that CDI and CDF had different mechanisms, but that CaM binding to the IQ motif is involved in both cases.

Ca_v_1.2 channels are constitutively associated with apo-CaM. Such pre-association is required since CaMs from the cytosolic bulk are unable to adequately access the binding site on Ca_v_1.2 during Ca^2+^ inflow (38); pre-association to the C-terminal region of Ca_v_1.2 places CaM within a nanodomain at channel cytosolic mouth. This location confers to the C-lobe of pre-bound CaM the ability to sense Ca^2+^ changes in temporal relation to channel gating (14).

#### CDI Mechanism

**Figure 2:**
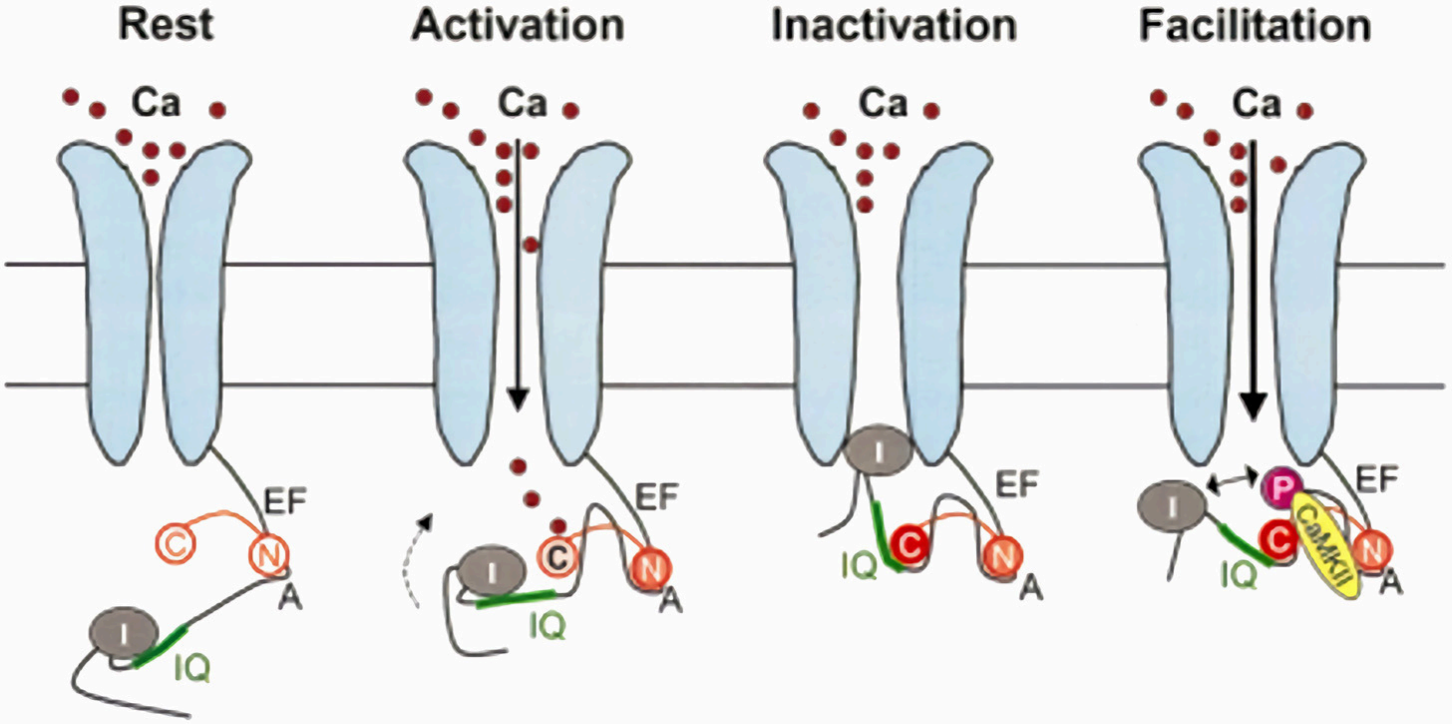
Model for CaM-dependent modulation of Ca_v_1.2 channels (I_CaL_). *CDI mechanism*: in the channel closed state (Rest), the N-lobe of apo-CaM (N) is constitutively bound to a pre-IQ region (A) in the channel C-terminus. When the channel opens (Activation), the CaM C-lobe (C) binds to the entering Ca^2+^, which increases its affinity for the channel IQ-domain; this moves the channel inactivation particle (I) in the permeation path (Inactivation). *CDF mechanism*: holo-CaM binding to CaMKII promotes channel phosphorylation, which results in repulsion of the inactivation particle from the permeation pore (Facilitation). Modified from Maier and Bers (8).

According to a recent interaction model, apo-CaM is constitutively tethered (pre-bound pool) to a pre-IQ motif present on the C-terminus of the α_1C_ channel subunit. Such “pre-association” involves the N-lobe and occurs at resting Ca^2+^ concentrations. Signaling activation by Ca^2+^ elevation (i.e., CDI induction) requires Ca^2+^ binding to the C-lobe, whose affinity for the target IQ motif is thus increased; C-lobe interaction with the IQ motif stabilizes the channel conformational state associated with CDI (8) (Figure 2). CDI has been further modeled as transitions between different states: apo-CaM release from the pre-association site, formation of the Ca^2+^-CaM complex, its subsequent binding to the effector site (14). The novel and most relevant feature of this model is that, using the difference in the kinetics of Ca^2+^ binding and unbinding between the C- and N-lobes (faster for the N-lobe), identifies their respective role in sensing Ca^2+^ at the channel mouth (local sensing, largely insensitive to intracellular Ca^2+^ buffering) vs. global cell Ca^2+^ (sensitive to even weak Ca^2+^ buffering). Unlike in neuronal channel isoforms, Ca_v_1.2 channels retain robust CDI even in the presence of strong Ca^2+^ buffering; such form of CDI is entirely triggered by Ca^2+^ association with the C-lobe (39). Numerical modeling provides the (counterintuitive) conclusion that, if associated with slow CaM-channel interaction kinetics, fast Ca^2+^ binding/unbinding (typical of the N-lobe) may best support selective sensing of a smaller but sustained Ca^2+^ signal (14). The latter is typical of CDI in non-cardiac channel isoforms (39).

#### CDF Mechanism

As discussed above, earlier studies indicated that CDF requires an intact anchoring region on the channel C-terminus, thus suggesting that pre-bound CaM is involved (37). Nonetheless, there is now general agreement that, unlike CDI, CDF is operated by Ca^2+^-CaM dependent activation of calmodulin-kinase II (CaMKII), which then phosphorylates the Ca_v_1.2 channel at two serine residues close to the EF-hand motif (40) (Figure 2); mutation of these two serine residues abolished CDF but did significantly affect CDI (40), hence confirming independent mechanisms for these processes. Notably, CaMKII phosphorylation of nearby serine residues also induces Mode2 gating of the channel (41). Thus, at variance with CDI, CDF is the consequence of protein phosphorylation, dependent on CaM, but not directly operated by it.

### Voltage-Gated K^+^ Channels (K_v_7.1)

K_v_7.1 is a K^+^-selective channel which, in association with KCNE subunits, carries the slow component of the delayed-rectifier current (I_Ks_). I_Ks_ gating is positively regulated by cytosolic Ca^2+^ through a CaM-dependent process (42), with an effect similar to that of the membrane constituent phosphatidylinositol-4,5-bisphosphate (PIP2). Indeed, PIP2 and the N-lobe of CaM competitively interact at the same site on the K_v_7.1 protein (the helix B on the proximal C terminus). Interpretation of the effect of a helix B K_v_7.1 mutant (K526E) and of interference of Ca^2+^-CaM with K_v_7.1 pull-down by PIP2 has led to the following model: at diastolic Ca^2+^ levels, CaM is bound to a non-activating K_v_7.1 site (helix A) by its apo-C-lobe; the N-lobe is displaced from the helix B site. As cytosolic Ca^2+^ increases, calcification of the C-lobe causes its dissociation from helix A and the N-lobe then interacts with its site on the helix B; this results in stabilization of the channel open state (43). According to an alternative model, at resting Ca^2+^ levels the C- and N-lobes are permanently bound to the channel (at helices A and B, respectively) and limit its open probability; when Ca^2+^ increases, N-lobe binding is reinforced and C-lobe is released thereby relieving the inhibitory effect on channel gating (44).

Both models imply that CaM binding to K_v_7.1 and positive regulation of I_Ks_ are separate processes. Binding occurs in the apo-CaM form (pre-bound pool), I_Ks_ enhancement requires Ca^2+^ elevation. Furthermore, both CaM lobes are involved and a preserved C-lobe Ca^2+^ affinity is essential for the signaling function.

PIP2 is a membrane phospholipid degraded by phospholipase C (PLC) to produce inositol 3-phosphate (IP3) in response to activation of a number of membrane receptors associated with G_q_ proteins. Receptor activation may result in PIP2 depletion, which would reduce I_Ks_; however, IP3-induced Ca^2+^ release from the sarcoplasmic reticulum may compensate PIP2 reduction by activating positive I_Ks_ regulation by Ca^2+^-CaM. This may represent the main physiological role of I_Ks_ modulation by Ca^2+^-CaM, which would therefore be of particular relevance during activation of the PLC-IP3 signaling pathway.

CaM integrity may also be necessary for K_v_7.1 channel trafficking; indeed, mutations disrupting N- and C-lobe integrity reduce channel membrane expression (44).

It has been reported that holo-CaM complexing with KCNE4 (channel β-subunit) inhibits I_Ks_ (45). While this would provide antagonism to direct holo-CaM modulation of the channel α-subunit, the physiological role of CaM-KCNE4 interaction remains unclear.

CaM-mediated I_Ks_ regulation also occurs indirectly by CaMKII-mediated phosphorylation of the channel at serine 484; contrary to direct CaM modulation, phosphorylative modulation is inhibitory and may account for I_Ks_ downregulation upon sustained β-adrenergic receptor activation (46).

### Voltage-Gated Na^+^ Channels (Na_v_1.5)

Among all voltage-gated channels involved in arrhythmogenesis, Na_v_1.5 channels also interact with CaM. CaM binds to an IQ motif on the C-terminus of this channel in a Ca^2+^-independent manner. Binding reduces CaM's affinity for Ca^2+^ and does not induce the conformational changes that have been observed for Ca_v_1.2 channels; therefore, similarities in the binding site may not necessarily translate into similarities of channel modulation. Nonetheless, disruption of the CaM binding site (by mutation of the Na_v_1.5 IQ motif) leads to the enhancement of persistent Na^+^ current, thus suggesting a role of CaM in stabilizing the inactivated state (47), possibly by fostering the interaction between the channel C-terminus and the II-IV linker. According to another model, CaM binding to Na_v_1.5 channels would obstruct their direct modulation by Ca^2+^; holo-CaM would lose its affinity for the channel, thus unveiling the direct modulatory site (48). In this case, failure of CaM interaction with the channel (as in the case of holo-CaM) causes a “leftward” (negative) shift of the steady-state inactivation curve (48); this would presumably reduce channel availability at diastolic potential as well as the “window” component of I_Na_.

Overall, while CaM interaction with Na_v_ and Ca_v_ channels are somewhat similar, the consequences of CaM-dependent modulation on Na_v_ function are less defined and, possibly, quantitatively less important.

## RyRs Modulation by Ca^2+^ and Ca^2+^-CaM

RyRs are homotetramers of ~2,200 kDa (each subunit is >550 kDa), containing ~5,035 amino acid residues in total, sharing the general structure of the six-transmembrane ion channel superfamily (49). Since RyRs span the SR membrane, they have domains facing both the cytosol and the SR lumen. Of the three isoforms present in nature, RyR2 is the predominant one in cardiac myocytes, where it is organized in large clusters on the SR membrane (50). In T-tubules, RyR2 clusters are separated from sarcolemmal Ca_v_1.2 channels by a 10–15 nm gap; thus, small Ca^2+^ influx through I_CaL_ exposes them to very high Ca^2+^ concentrations (50, 51). This structural arrangement is generally referred to as “couplon” (52). The RyR/Ca_v_ ratio in couplons is up to 15-fold higher in cardiac than in skeletal muscle and differs between species (53).

RyRs are strongly regulated by Ca^2+^ in CaM-independent ways. At the same time, they are regulated by CaM in both Ca^2+^-dependent and -independent ways. This makes investigation of CaM's role in RyRs' regulation very complex.

### CaM-Independent RyR Regulation by Ca^2+^

RyR gating is highly sensitive to Ca^2+^ on both sides of the SR membrane in a CaM-independent way. Although not the focus of the present review, a brief discussion of such “direct” regulation by Ca^2+^ is required to understand the potential difficulty in isolating the CaM-dependent one.

Each of the N- and C-terminal domains of RyR2 contains two EF-hand Ca^2+^ binding motifs (54), similar to those of CaM. These motifs are both on the cytosolic domain of RyR2; they show high (C-terminal) and low (N-terminal) affinity for Ca^2+^ and induce channel opening and inactivation, respectively (55, 56). Since RyR2 inactivation occurs at Ca^2+^ concentrations exceeding the physiological range, Ca^2+^-dependent activation is the dominant phenomenon and the basis for the Ca^2+^-induced-Ca^2+^release (CICR) mechanism (7).

SR luminal Ca^2+^ modulates RyR2 open probability by two CaM-independent mechanisms. The indirect one involves stabilization of the closed state by a macromolecular complex, involving calsequestrin (CASQ) and is disrupted by increases in luminal Ca^2+^ (57). Sensitivity to luminal Ca^2+^ is preserved after CASQ knock out. This stands for the presence of a Ca^2+^-sensing mechanism on the RyR protein itself, located in a luminal domain also involved in control of Ca^2+^ permeation (58). Direct lumenal Ca^2+^ sensing may be important for channel activation under conditions of SR overload (59).

### CaM-Dependent RyR Regulation

CaM-dependent modulation of RyRs differs between channel isoforms, which have only ~70% of gene homology and contain three divergent regions (60). In general, conductance of all the three RyR isoforms is reduced by CaM when cytosolic Ca^2+^ is above 1 μM. At lower Ca^2+^ concentrations, favoring apo-CaM and more relevant to diastole, CaM increases the open probability of RyR1 and RyR3 (61, 62), but it stabilizes the closed conformation of RyR2 (54).

RyR2 channels have high affinity (nanomolar K_d_) for both apo-CaM and holo-CaM, thus resulting in a pre-bound CaM pool (54, 63). Apo-CaM may actually be a stronger inhibitor of RyR2 opening than holo-CaM, as indicated by relief of inhibition at Ca^2+^ concentrations in the μM range (64). Therefore, CaM modulation of RyR2 gating may be largely Ca^2+^-independent. Increasing Ca^2+^ up to 100 μM has been reported to increase the number of CaM molecules bound to RyR2 (from 1 to 7.5 per RyR2 tetramer) (54); however, it is difficult to relate responses to such an abnormally high Ca^2+^ concentration to physiological function.

Given that CaM binding domains are highly homologous between RyR1 and RyR2 (65), what may explain the Ca^2+^-dependent discrepancy of CaM effects on RyR opening between RyR1 and RyR2 channels?

**Figure 3:**
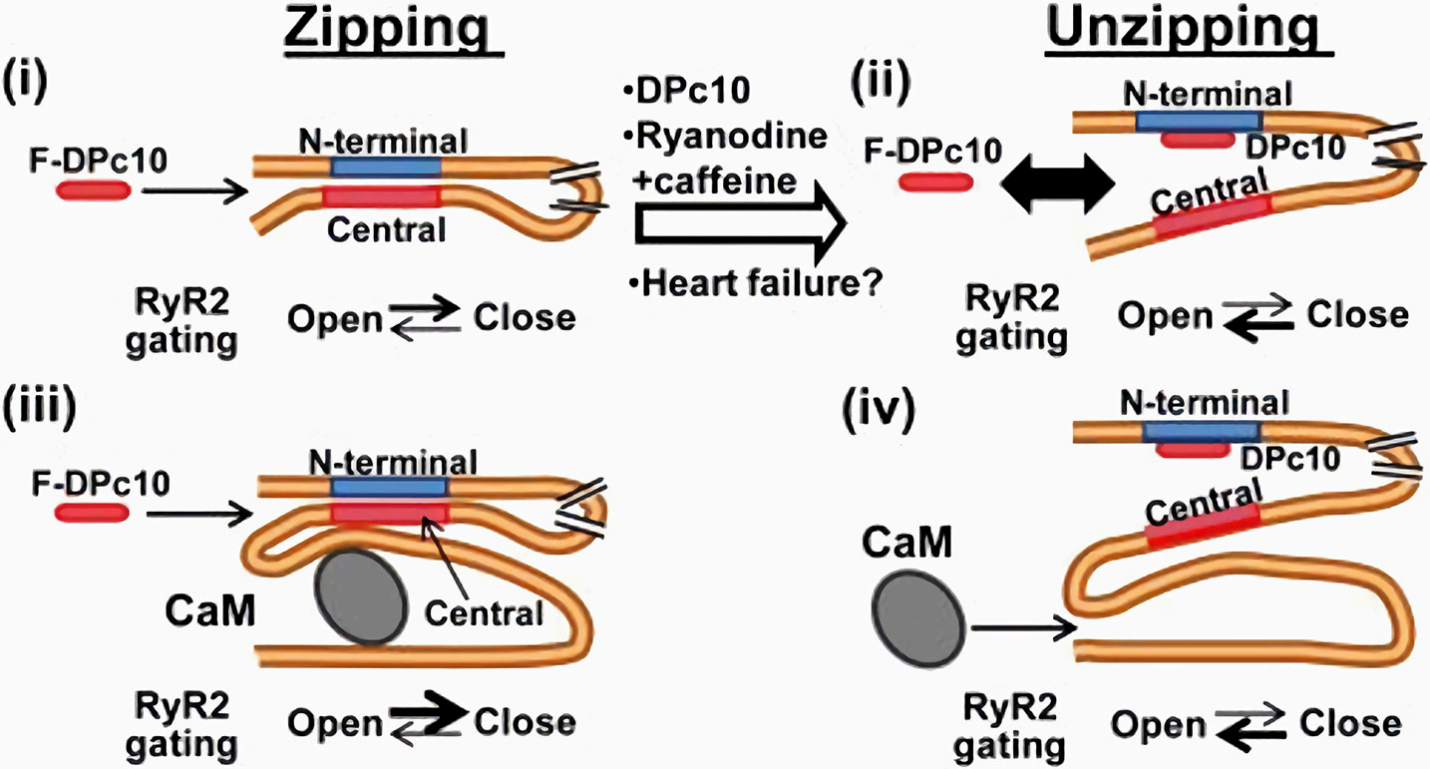
CaM-dependent modulation of RyR2 channels. RyR2 closed state is stabilized by the interaction (zipping) between “terminal” and “central” regions of the N-terminal (cytosolic) tail of the protein. If such interaction is removed (unzipping), the channel closed state is destabilized. Apo-CaM binds to a domain distal to the “zipping” one, but the resulting conformation allosterically facilitates the zipping interaction, thus stabilizing RyR2 closed state. CaM and F-DPc10 (a peptide obstructing the zipping interaction) allosterically “compete” for binding to RyR2. Similarly, the unzipped state, promoted by drugs and reactive oxygen species which facilitate RyR2 opening, reduces RyR2 affinity for CaM (67). F-DPc10 is a peptide fragment designed to prevent the interaction between the central and N-terminal protein domains (a tool in testing the unzipping model). From Oda et al. (66).

Mutations in both the N-terminal and central RyR2 regions similarly destabilize the channel closed state; this suggests that the latter may require interaction between these two regions (“zipping” model of RyR gating). This view is confirmed by the effect of peptides (e.g., DPc-10) interfering with such interaction (66). On the other hand, CaM binds to a domain other than those involved in zipping. According to the “inter-domain hypothesis,” CaM binding to RyR2 may induce a protein conformation change that allosterically stabilizes the zipping interaction (and the closed state) (Figure 3). Vice versa, agents interfering with the zipping interaction may reduce CaM binding affinity (66).

A different gating model has been proposed for RyR1 channels, in which the channel CaM binding domain is followed, within about 450 residues, by a CaM-like sequence. FRET data indicate that, at high Ca^2+^, the two channel domains interact with each other in the folded protein; such Ca^2+^-dependent interaction is required for channel opening. Binding of holo-CaM to the channel may disrupt the activating interdomain interaction, thus explaining CaM-induced RyR1 inhibition at high Ca^2+^ concentrations (68). However, this model does not explain why low Ca^2+^ concentrations (favoring apo-CaM) may produce CaM-dependent RyR1 activation, which remains an open question.

Cryo-EM studies in RyR1 indicate that increasing Ca^2+^ shifts CaM binding to the channel by about 3 nM, corresponding in the 3D structure to two different protein domains. In view of the fact that CaM activates RyR1 at low Ca^2+^ and inhibits it at high Ca^2+^, the two domains may be seen as activator and inhibitory sites, respectively. Importantly, these studies indicate that the Ca^2+^-dependent shift in CaM binding site is a consequence of a rearrangement of the binding surface of CaM, rather than of RyR1 conformation (69) (which is also Ca^2+^-dependent). The same technique shows that in RyR2 CaM binds to the “inhibitory site” (as identified in RyR1) already at low Ca^2+^; this might explain why RyR2 is inhibited by CaM in a Ca^2+^-independent way (69). Apparently at odd with these observations, FRET experiments (in the same study), measuring the position of CaM relative to that of FKBP in RyR1, did not detect Ca^2+^-dependent shifts in CaM position of a magnitude compatible with the results of cryo-EM data (69). However, Ca^2+^-induced structural changes in both probe-carrying proteins are possible and might minimize FRET distances even in the presence of real shifts in binding position.

In conclusion, even if the detailed mechanism remains to be resolved, it is now accepted that direct regulation of RyR gating by CaM is Ca^2+^-dependent (stimulatory to inhibitory) in RyR1 and Ca^2+^-independent (always inhibitory) in RyR2. Formation of the Ca^2+^-CaM complex does affect RyR2 gating significantly, but this occurs through an indirect mechanism, involving CaMKII activation.

CaMKII is a cytosolic serine-threonine kinase activated by Ca^2+^ with a K_d_ of 20-100 nM, which dramatically decreases (to 60 pM) after enzyme autophosphorylation (8). The Ca^2+^-CaM complex activates kinase activity by binding to an enzyme regulatory region, located in the central protein domain. Enzyme activation occurs by displacement of an auto-inhibitory segment that occludes access of the substrate to the N-terminal catalytic domain. As for other targets, both N- and C-terminal CaM lobes are involved in activation of kinase activity (25), which is in this case strictly Ca^2+^-dependent.

CaMKII mediates a number of Ca^2+^-activated phosphorylations, including that of RyR2 at Ser2814 on the cytosolic surface of the channel (exclusively for CaMKII) and, additional serine residues (49, 70). Most of the evidence converges to show that CaMKII-dependent phosphorylation facilitates opening of RyR2 channels, thus increasing sensitivity of SR Ca^2+^-release to cytosolic Ca^2+^. This may be particularly relevant under pathological conditions (70).

CaM binding to RyRs also depends on factors other than Ca^2+^, such as pH, Mg^2+^ oxidation state (54, 62). In particular, oxidative modifications compromise the normal activity of RyR2 by influencing their luminal Ca^2+^ regulation in a manner similar to that observed in heart failure (71).

Stabilization of RyR2 closed state by CaM is crucial in minimizing spontaneous (non-triggered) Ca^2+^ release from the SR in the form of either “Ca-leak” (random release from individual RyRs) or “Ca^2+^ spark” (synchronous release from a RyR2 cluster), a function pivotal to both contractile and electrical function of cardiomyocytes (63).

## Phenotypes in CaM Mutations and Underlying Mechanisms

Mutations in one of the three CALM genes, even in the heterozygous form, have been described in patients with a severe cardiac phenotype, characterized by a high propensity to ventricular arrhythmias, syncopal episodes and sudden death at a young age (72).

Despite sharing strong electrical instability, two distinct phenotypes can be identified in carriers of CaM mutations: the long QT syndrome (LQTS) (9, 73), characterized by prolongation of repolarization, and catecholamine-induced ventricular tachycardia (CPVT), characterized instead by exercise-induced ventricular arrhythmias (74). In general, each specific CALM mutation is associated with one of the two phenotypes; nonetheless, mutations with mixed phenotypes have also been described (72). Such confounding complexity contrasts with an apparently sharp separation of the molecular mechanisms underlying the LQTS and CPVT patterns. A third, less well-defined arrhythmia phenotype, idiopathic ventricular fibrillation (IVF), has also been associated with a CALM mutation (75) and will be addressed here in paragraph Mixed phenotype.

A point of interest in the interpretation of CALM mutations is their extremely high penetrance: 1 mutant allele in 6 encoding for the same amino acid sequence (as in heterozygous mutations) is sufficient to result in marked functional derangements.

Also in view of the multiplicity of functions exerted by CaM in many cell types, all this suggests that, for one reason or another, mutant CaMs must interact with their target with high specificity. In the following paragraphs we will describe the cellular functional derangements associated with the LQTS and CPVT phenotypes and address, as much as current knowledge allows, the mechanisms underlying target specificity of CaM mutations.

### LQTS Phenotype

Prolongation of action potential duration (APD), reflected as QT interval prolongation on the ECG, can result from loss of function of outward currents, or gain of function of inward ones; therefore, CaM abnormalities affecting modulation of K_v_7.1 (I_Ks_) and Ca_v_1.2 (I_CaL_) might theoretically be involved in prolongation of repolarization. Enhancement of the “window” (I_NaW_) or “late” (I_NaL_) components of the Na^+^ current I_Na_ (Na_v_1.5 channel) might represent a further potential mechanism.

Nevertheless, gain of Ca_v_1.2 function (I_CaL_ enhancement) has emerged as the dominant mechanism in CALM gene mutations associated with delayed repolarization.

In 2013, Crotti et al. reported three *de novo* heterozygous missense CALM gene mutations in LQT-infants with recurrent cardiac arrest (73). In particular, the *CALM1*-p.D130G and *CALM2*-p.D96V mutations affect highly conserved aspartic acid residues (C-lobe EF IV and EF III, respectively) involved in Ca^2+^ binding. The *CALM1*-p.F142L (next to C-lobe EF IV), albeit outside the EF-hand, is expected to alter the energetics of the conformational change associated to Ca^2+^ binding (22). The p.D130G mutation, associated with the LQTS phenotype, has also been identified in the *CALM3* gene (76), thus reinforcing the concept that mutation effect may be independent of the gene affected. *In vitro* Ca^2+^ binding studies revealed that all these three mutations are characterized by a 5- to 50-fold reduction in Ca^2+^ binding affinity of the C-lobe, without affecting N-lobe affinity (73). Overexpression of these mutations in guinea-pig myocytes or an engineered cell line showed loss of I_CaL_ CDI, leading to action potential prolongation with enhanced intercellular variability. The amplitude of Ca^2+^ transients and its dispersion were also increased, likely secondary to increased Ca^2+^ influx; notably, spontaneous Ca^2+^ release events were not reported (77). Indeed, consistent with the LQTS phenotype, RyR2 function was unaffected. Binding of CaM mutants to Ca_v_1.2 was tested by FRET and found to be enhanced for p.F142L and unaffected by the other mutations. On the other hand, titration of WT vs. mutant expression levels showed that a ratio (WT/mutant) of 7 (compatible with heterozygosity) was enough to impair CDI (77). Therefore, selectivity of mutant CaMs in altering Ca_v_1.2 channel function can be explained by the fact that modulation of this target requires a pre-bound apo-CaM pool (containing mixtures of WT and mutant CaMs) and subsequent Ca^2+^ binding to this pool (impaired in mutants by loss of Ca^2+^ affinity). This interpretation would explain sparing of RyR2, whose modulation may not require Ca^2+^ binding, and of CaMKII, which binds CaM directly in its holo-form (not represented if Ca^2+^ affinity is reduced). Selective CDI impairment by additional mutations reducing C-lobe Ca^2+^ affinity (*CALM2*-p.D132H and *CALM1*-p.D132V) has also been reported in transfection studies on human induced pluripotent stem cell-derived cardiomyocytes (78).

Other heterozygous LQTS mutations, *CALM2*-p.D130V and *CALM1*-p.E141G have been recently identified by Boczek et al. (79). As in p.D130G, the former involves replacement of aspartic acid by a neutral residue; therefore, loss of C-lobe Ca^2+^ affinity is to be expected. *CALM1*-p.E141G has a phenotype closely resembling that of *CALM1*-p.F142L, indicating that residues 141 and 142 are both crucial for C-lobe Ca^2+^ binding. Notably, when transfected in isolation, *CALM1*-p.E141G also enhanced I_NaL_, but the effect disappeared when mutant and WT constructs were co-expressed. This is consistent with a role of CaM stabilizing Na_v_1.5 inactivation (see above) and implies lack of functional dominance of the mutation for this target. However, at least according to a current model of CaM-Na_v_1.5 interaction (48), reduced affinity of mutant CaM for Ca^2+^ would not explain I_NaL_ enhancement.

CaM mutations resulting in downregulation of K^+^ currents have not been reported, even if I_Ks_ function has been tested in some cases (*CALM1*-p.F142L) (80). Nonetheless, the K_v_7.1 α-subunit mutation p.K526E, accounting for a case of LQT1, impairs interaction of the channel's helix B with CaM's N-lobe. This leads to I_Ks_ downregulation and delayed repolarization (43). Considering that the mode of CaM–K_v_7.1 interaction involves a pre-bound pool and Ca^2+^-dependent C-lobe signaling (as for Ca_v_1.2), it is surprising that CaM mutations with loss of C-lobe Ca^2+^ affinity may not affect I_Ks_. One tentative explanation is compensation of the loss of CaM-dependent regulation by PIP2 signaling; it would be therefore interesting to evaluate the effect of known CaM mutations on I_Ks_ under conditions of PIP2 depletion.

### CPVT Phenotype

Catecholaminergic polymorphic ventricular tachycardias are malignant arrhythmias with an ECG pattern suggesting multifocal origin (unlike TdP), typically induced by exercise, or other conditions associated with enhanced adrenergic stimulation (81). The prototypical form of this arrhythmia has been associated with mutations of RyR2 channels, or of the proteins associated with them in a macromolecular complex (junction, triadin, calsequestrin, sorcin etc.). The electrical disturbance at the basis of CPVT originates from “Ca^2+^ waves,” i.e., macroscopic surges of cytosolic Ca^2+^ resulting from spontaneous RyR opening at a point site, followed by auto-regenerative propagation (by Ca^2+^-induced Ca^2+^ release) of the ionic perturbation to the whole cell (82). The mechanism connecting the Ca^2+^ wave to membrane potential is Ca^2+^-induced activation of the electrogenic Na^+^/Ca^2+^ exchanger (NCX), which results in a depolarizing current, also referred to as “transient inward current” (I_TI_). While “Ca^2+^ overload” facilitates Ca^2+^ waves (by increasing RyRs open probability), it is neither necessary nor sufficient to induce them; indeed some degree of intrinsic RyR2 instability may be involved even in the prototypical case of digitalis toxicity (83).

CaM mutations associated with the CPVT phenotype are generally characterized by a relatively small impairment of C-lobe Ca^2+^ binding (e.g., *CALM1*-p.N98S, *CALM1*-p.N54I, and *CALM3*-p.A103V) (84, 85), which (as shown for *CALM3*-p.A103V) (85) corresponds to a minor effect on I_CaL_ CDI. Besides this, the relationship between mutation features and the CPVT phenotype is somewhat elusive. Nyegaard and colleagues tested *CALM1*-p.N54I and -p.N98S binding to a small RyR2 segment, found a decrease in *CALM1*-p.N98S affinity only at low Ca^2+^ levels and explained mutation phenotype with loss of CaM-RyR2 complexing. These findings were later contradicted by studies using the entire RyR2 protein (86), that detected an increase in RyR2 affinity for both these mutations (also accounting for dominance of effects). Nonetheless, increased affinity for RyR2 may not be a prerequisite for channel destabilization; indeed the CPVT mutation *CALM3*-p.A103V, which strongly increased Ca^2+^ release events, displayed normal RyR2 affinity (85). Notably, mutations sites N54 and N98, albeit affecting the N- and C-lobes, respectively, are not contained within any known protein-protein interaction sites (84).

In conclusion, the features of CPVT mutations may explain why they are not generally associated with an LQTS phenotype (however see below), but a general mechanism by which they induce RyR2 instability cannot be clearly envisioned. Since CaM interaction with RyR2 is essentially Ca^2+^-independent, the mechanism must conceivably reside in a change of 3D protein conformations involved in CaM-RyR2 complexing; nonetheless, the nature of this change remains to be clarified.

### Mixed Phenotype

Notwithstanding the apparently sharp demarcation of CaM abnormalities affecting Ca_v_1.2 and RyR2 channels, several mutations have been associated with both LQTS and CPVT phenotypes. CaM mutations were found in five subjects with QT prolongation; nonetheless, two of them (*CALM2*-p.D132E and –p.Q136P) were associated with arrhythmia features strongly suggestive of SR instability and thus were assigned to the CPVT phenotype (72). These mutations affect EF III and EF IV of the C-loop and displayed reduced Ca^2+^ affinity; thus, even if I_CaL_ CDI was not directly tested in this study, it may be tentatively considered responsible for the observed QT prolongation. Intriguingly, two different reports assign LQTS and CPVT phenotypes to the same mutation (p.N98S) occurring in genes *CALM2* (72) and *CALM* (84), respectively; since the CaM amino acid sequence encoded by the 2 genes is identical, other factors should account for the discrepancy.

The reason for SR instability in all these cases is as elusive as the properties of mutations that favor RyR2 dysfunction (see above). Nonetheless, it should be considered that impairment of I_CaL_ CDI and the resulting APD prolongation are obviously stress conditions for intracellular Ca^2+^ homeostasis, requiring robust compensatory mechanisms. Thus, albeit not observed in hiPSC-CMs from an LQTS case (80), SR instability secondary to defective CDI might occur in subjects (or conditions) in which such compensation is less efficient. Thus, assignment to an LQTS or CPVT clinical phenotype may not be always accurate in defining the mutation-induced abnormality accounting for arrhythmogenesis.

Marsman et al. have described the *CALM1*-p.F90L mutation in a patient with IVF, i.e., VF episodes without the features of either LQTS or CPVT (mild QT prolongation only during exercise recovery) (75). The mutation, which resides on the linker between EF III and EF IV, impairs C-lobe Ca^2+^ affinity conformational stability and CaM-RyR2 interaction (87). In heterologous expression experiments, p.F90L also affects small-conductance Ca^2+^-activated K^+^ channels (SK channels) (88); nonetheless, the role of these channels in ventricular electrophysiology is unclear.

## Evaluating CaM Mutations in Patient-Derived Cardiomyocytes

Induced pluripotent stem cell-derived cardiomyocytes from mutation carriers (hiPSC-CMs) provide the means to test mutation effects in the context of each patient's genetic background. Recent studies have exploited this cellular model to test the effect of CaM mutations. Yamamoto et al. reported a typical LQTS phenotype for the heterozygous *CALM2-*p.N98S mutation and obtained reversal of the phenotype by knocking out the mutant allele by gene editing (89), thus supporting a causal relationship between mutation and phenotype. Notably, *CALM2-*p.N98S affinity for Ca^2+^ is only mildly reduced, and a CPVT phenotype has also been reported for this mutation (84).

**Figure 4:**
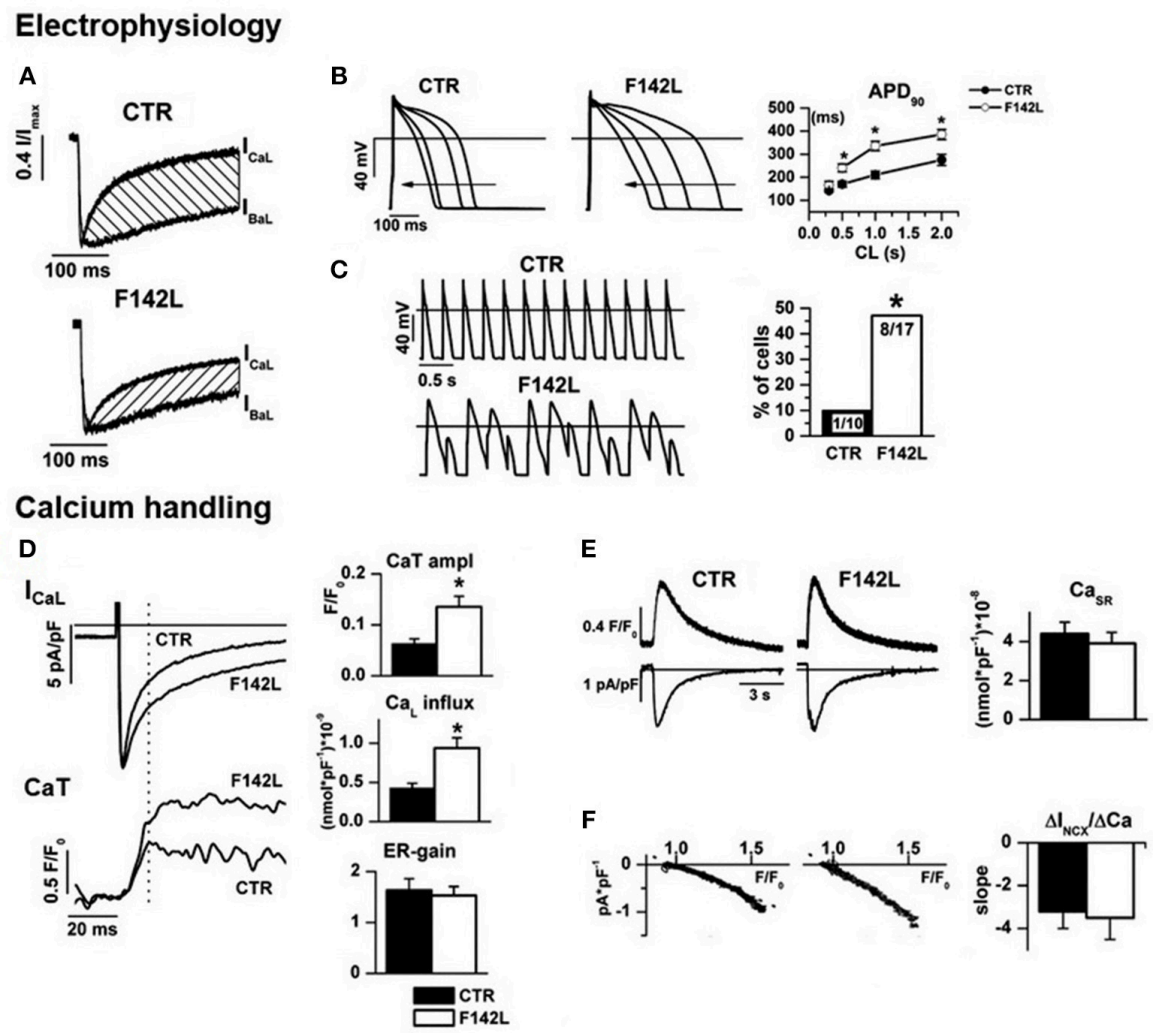
Arrhythmogenic mechanism of CALM1 F142L from experiments in patient-derived hiPSC-CMs. *Electrophysiology:* **(A)** I_CaL_ CDI (hatched area) was reduced; **(B)** CDI impairment led to APD prolongation and inadequate APD shortening at high pacing rate; **(C)** APD abnormalities led to loss of 1:1 response to fast pacing in a large % of F142L cells. *Calcium handling*: **(D)** Impaired I_CaL_ CDI led to matching increments of Ca^2+^ influx and of the amplitude of Ca^2+^ transients (CaT); excitation/release gain (ER-gain) was unchanged, thus suggesting normal RyRs function. **(E)** In spite of enhanced Ca^2+^ influx, SR Ca^2+^ content was unchanged, thus implying compensation by homeostatic mechanisms. **(F)** The slope of the relationship between Na^+^/Ca^2+^ exchanger current (I_NCX_) and Ca^2+^ concentration was unchanged, to indicate that homeostatic compensation did not involve changes in the expression of the exchanger. Asterisks denote significance of changes. Modified from ref. Rocchetti et al. (80).

We recently investigated hiPSC-CMs from a patient with LQTS phenotype and heterozygous carrier of the *CALM1*-p.F142L mutation (80) (Figure 4). CDI of I_CaL_ was severely impaired, thus accounting for APD prolongation (which was reversed by I_CaL_ blockade) and its failure to shorten adequately at high pacing rates. As expected from the increase in Ca^2+^ influx, the amplitude of V-triggered Ca^2+^ transients was significantly increased; nonetheless, SR Ca^2+^ content was normal and no spontaneous Ca^2+^ release events were observed, thus suggesting preserved homeostatsis of intracellular Ca^2+^. This argues against SR instability as the arrhythmogenic mechanism in this case and suggests a primary role of prolonged and “stiff” (non-rate-adaptive) repolarization instead (80). Other currents under CaM modulation were also evaluated in this study: I_Ks_ was found to be unaffected and a persistent component of I_Na_ (likely contributed by I_NaW_) was significantly reduced (80). While this confirms loss of I_CaL_ CDI as the sole mechanism of repolarization abnormality, I_NaW_ reduction was unexpected; indeed, loss of CaM affinity for Ca^2+^ should if anything, increase I_NaW_ (48). Notably, CaMKII activity was preserved and even slightly enhanced, probably secondary to the increase in Ca^2+^ transients amplitude. This supports the view that negative dominance of the mutation only applies to targets, as Ca_v_1.2, binding CaM in its apo form.

Altogether, these findings clearly confirm loss of I_CaL_ CDI as the mechanism underlying the LQTS phenotype in CaM mutations with reduced Ca^2+^ affinity. To our best knowledge, no hiPSC-CMs studies are thus far available for mutations with a clear-cut CPVT phenotype. It should be considered that, due to immaturity of the structures involved in intracellular Ca^2+^ handling (e.g., lack of T-tubules) (90), hiPSC-CMs may be less suitable in evaluating CaM mutations leading to SR instability (CPVT phenotype).

## Conclusions and Therapeutic Implications

CaM functions as a Ca^2+^ sensor to maintain physiological Ca^2+^ levels in cells. In addition to this homeostatic role, CaM signal targeting is required to transduce fundamental cell processes, for which Ca^2+^-CaM complexing is not necessarily involved, but may still have a modulatory effect. This is possible because of the presence of CaM-binding sequences suitable to allow CaM to bind multiple targets even in its “apo” form; this generates a quantitatively prevailing “pre-bound” CaM pool. CaM binding to targets occurs with very high specificity, which is required to explain restriction of CaM mutations phenotype to the myocardium and, within it, to specific subcellular targets.

Whereas mutation-induced loss of Ca^2+^ sensing function is crucial in impairing CDI of sarcolemmal Ca^2+^ channels (carrying I_CaL_), it is not required for mutations associated with RyR2 instability. For the latter, changes in CaM affinity for RyR2 channels are apparently more important; however, the direction and even the need for such changes are still unclear. Possibly, mutations induce complex (3D) modifications in the protein-protein binding interface, of which changes in CaM affinity for the target are just a gross readout. This is a field in which new information is strongly required.

Whereas, “pure” LQTS and CPVT phenotypes suggest abnormal modulation of I_CaL_ and RyR2, respectively, we hypothesize that coexistence of QT prolongation and SR instability (mixed phenotypes) might be accounted for by impaired I_CaL_ CDI, possibly with the complement of (very common) conditions weakening homeostatic control of intracellular Ca^2+^.

Based on the information reviewed above, mechanism-guided therapeutic approaches to calmodulinopathies should ideally address the interaction of mutant CaM with its targets. Particularly in the case of LQTS-type mutations, this approach is justified by the role of the high target affinity of mutant CaMs in causing negative dominance of the mutation. Tools for this purpose are not available yet, but possibilities exist and are currently explored.

Therapy of CaM mutations with more classical approaches may depend on the phenotype. I_CaL_ blockade seems a logical approach in the case of I_CaL_ gain of function, resulting from loss of CDI (LQTS phenotype); indeed, verapamil did shorten the QT interval in hiPSC-CMs from *CALM1*-p.F142L carriers (80). Nonetheless, selective inhibition of the sustained I_CaL_ component would be desirable and should be pursued by developing I_CaL_ blockers with such a property; as suggested by availability of selective blockade of I_Na_ sustained component (91), this should be seen as an achievable goal. Pharmacological treatment of CPVT-type CaM mutations may require RyR2 stabilization, or at least, blunting membrane electrical response to spontaneous Ca^2+^ release events. This is a long-pursued goal for which no ideal tool has been thus far identified; while agents as flecainide or carvedilol may provide some protection [for review see Zaza and Rocchetti (82)], the search of clinically usable specific RyR2 blockers is currently ongoing.

Because calmodulinopathies have been recently described, information on the clinical efficacy of therapies is not available yet.

## Future Challenges

Calmodulinopathies have undoubtedly attracted high quality, multidisciplinary research; nonetheless, and rather unsurprisingly, many questions have yet to be addressed. The present review highlights a few that, in our view, might deserve particular attention.

The role and mechanism of Na_V_1.5 dysregulation in CaM mutation-associated phenotypes is elusive. Notably, I_NaL_ enhancement, a target for which therapeutic interventions are available, might have a role in both QT prolongation (LQTS phenotype) and SR instability (CPVT phenotype) (91).

The interplay between CaM- and PIP2-dependent modulation of I_Ks_ suggests that factors affecting membrane PIP2 levels (e.g., phospholipase-C signaling) potentially influence CaM mutation penetrance. If this were the case, such factors might represent easily accessible therapeutic targets.

The ultimate mechanism of arrhythmia facilitation by loss of I_CaL_ CDI, which seems to diverge from what would be expected, has thus far been only superficially addressed.

Finally, the molecular basis of RyR2 dysfunction in the context of CaM mutations remains largely unresolved, thus preventing identification of mechanism-specific targets.

## Author Contributions

BB wrote a general manuscript draft. CR focused on the section about CaM modulation of voltage-gated channels. M-CK, LS, and AG provided text and discussion for integration with clinical and genetic aspects of calmodulinopathies. LC and AZ supervised the process and edited the manuscript to its final version.

### Conflict of Interest Statement

The authors declare that the research was conducted in the absence of any commercial or financial relationships that could be construed as a potential conflict of interest.
